# Effects of Aspirin Therapy on Bypass Efficacy and Survival of Patients Receiving Direct Cerebral Revascularization

**DOI:** 10.3389/fphar.2022.841174

**Published:** 2022-05-03

**Authors:** Yanxiao Xiang, Ping Zhang, Peng Zhao, Tao Sun, Fei Wang, Yiming He, Donghai Wang, Anchang Liu

**Affiliations:** ^1^ Department of Pharmacy, Qilu Hospital of Shandong University, Jinan, China; ^2^ Department of Neurosurgery, Qilu Hospital of Shandong University, Jinan, China

**Keywords:** moyamoya disease, aspirin, STA-MCA, bypass efficacy, postoperative complications

## Abstract

**Background:** Both patency maintenance and neoangiogenesis contribute to cerebrovascular bypass efficacy. However, the combined impact of the aforementioned two indicators on postoperative revascularization following superficial temporal artery-to-middle cerebral artery (STA-MCA) bypass has been less well elucidated. Meanwhile, there is a paucity of evidence with conflicting results about postoperative aspirin therapy.

**Objective:** The objective of the study was to investigate the correlation between aspirin use and STA-MCA bypass efficacy, including patency, postoperative neoangiogenesis, and follow-up outcomes.

**Methods:** A total of 181 MMD patients (201 procedures) undergoing STA-MCA bypass at our institution (2017–2019) were retrospectively reviewed. The bypass efficacy level and postoperative complications were compared between aspirin and non-aspirin groups.

**Results:** Among 95 PS-matched pairs, the aspirin group presented a significantly more favorable bypass efficacy than the non-aspirin group [odds ratio (OR) 2.23, 95% confidence interval (CI) 1.11–4.61; *p* = 0.026]. Multivariate logistic regression analysis confirmed the profound impact of aspirin as an independent predictor of bypass efficacy [adjusted OR 2.91, 95% CI 1.34–6.68; *p* = 0.009]. A remarkable negative correlation was found between bypass efficacy and the rate of ischemic complications (Phi = −0.521). Postoperative aspirin therapy was associated with a non-significant trend toward a lower incidence of ischemic events [OR 0.73, 95% CI 0.23–2.19; *p* = 0.580]. No significant difference in bleeding rates was observed between aspirin and control groups [OR 1.00, 95% CI 0.12–8.48; *p* = 1.000].

**Conclusion:** Among patients undergoing STA-MCA bypass procedures, bypass efficacy is a good predictor of follow-up outcomes. Postoperative aspirin therapy can improve patency, neoangiogenesis, and overall bypass efficacy, thereby protecting against postoperative ischemic complications.

**Clinical Trial Registration:** http://www.chictr.org.cn/, identifier CTR2100046178.

## Introduction

Moyamoya disease (MMD) is a chronic cerebrovascular disease characterized by progressive arterial stenosis and occlusion around the circle of Willis that results in cerebral ischemia as well as intracranial hemorrhage (Baba et al., 2008; Kuroda and Houkin, 2008). Although the pathogenesis of MMD is poorly understood, surgical revascularization plays a pivotal role in the management of affected individuals, allowing an increase in intracranial perfusion and a reduction in the burden of moyamoya vessels, and is associated with an improved prognosis (Kuroda and Houkin, 2008; Scott and Smith, 2009).

Currently, superficial temporal artery-to-middle cerebral artery (STA-MCA) bypass is widely regarded as the revascularization strategy of choice, particularly in adult patients with ischemic MMD (Zhao et al., 2017). However, postoperative ischemia has been reported to have an occurrence risk of up to 4.5% after STA-MCA bypass (Kim et al., 2016a; Zhao et al., 2018). In these studies, bypass efficacy is a key determinant of the success of the intervention and the long-term survival of the patients. Therefore, targeted follow-up measures of bypass efficacy are essential to predict the clinical prognosis. Bypass patency may be one factor related to STA-MCA bypass efficacy, affecting the occurrence of ischemic stroke during the follow-up period. Patients with bypass patency present a better neurological status than those with bypass occlusion throughout follow-up (Deng et al., 2018; Lu et al., 2021). In addition, collateral circulation angiogenesis may also play a role in clinical improvement (Kim et al., 2016b). When neoangiogenesis develops postoperatively, parameters reflecting the cortical hemodynamics improve and clinical symptoms improve or stabilize (Nariai et al., 1994; Schubert et al., 2012; Lu et al., 2021). However, the impact of postoperative neoangiogenesis among patients receiving a STA-MCA bypass procedure has not been well studied.

Aspirin has been administered as standard antiplatelet therapy after coronary bypass surgery to improve microcirculation, maintain blood flow through the bypass, and reduce mortality and major adverse cardiac event rates (Valgimigli et al., 20172018). However, because of the heterogeneity of MMD, the effects of antiplatelet agents after revascularization remain a matter of debate. Currently, some experts are in favor of aspirin therapy after surgical revascularization since it is associated with angiographic and clinical improvement for a prolonged period (Onozuka et al., 2016). Meanwhile, some data suggest that the overall benefit of aspirin therapy after bypass procedures for preventing recurrent stroke might be reduced due to an increase in the risk of brain hemorrhage (Kraemer et al., 2012).

Given our significant experience with cerebrovascular bypass that has been accumulated over recent years, we set out to explore the effects of aspirin on surgically managed patients with MMD. The aim of this study was to evaluate the impact of bypass patency and postoperative neoangiogenesis on the outcome of patients after STA-MCA bypass treatment as well as to investigate the correlation of aspirin treatment with bypass efficacy and clinical prognosis.

## Methods

### Study Design and Participants

**FIGURE 1:**
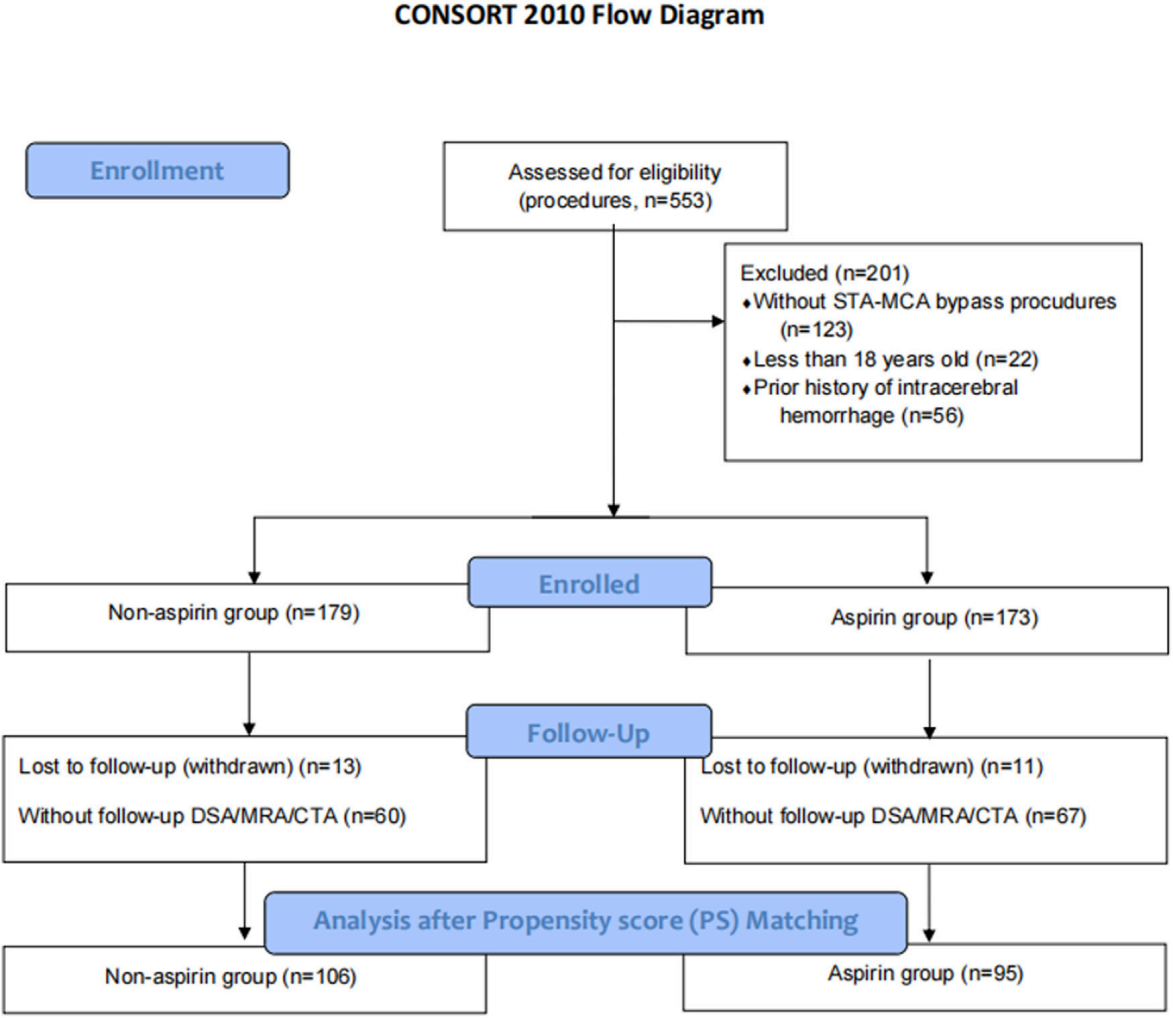
Flow diagram of the progress through the study.

This is a single-center retrospective registry cohort study on the effects of postoperative aspirin use for MMD. The study protocol was approved by the Institutional Review Board (IRB) of our institution, and the study was conducted in accordance with institutional ethical guidelines. The sample size was estimated by MedCalc 18.2.1 software. All MMD patients who were diagnosed using digital subtraction angiography (DSA) and/or MR angiography (MRA) and/or CT angiography (CTA) in the neurosurgical wards of our institution between 1 January 2017 and 31 December 2019 were screened (Figure 1) The diagnosis was based on the guidelines for MMD (criteria of the Research Committee on Spontaneous Occlusion of the Circle of Willis, 2012) (Research Committee on the Pathology, 2012). We excluded those who met the following criteria: 1) without STA-MCA bypass procedures, 2) less than 18 years old, 3) withdrawn, 4) without follow-up for DSA/MRA/CTA, 5) prior history of intracerebral hemorrhage, and 6) irregular and consistent aspirin intake. Informed consent was obtained from all patients.

### Baseline Data Collection

The baseline clinical variables examined were sex, age, smoking, alcohol consumption, past medical history, presenting symptoms, hospitalization days, length of follow-up, modified Rankin scale (mRS) score, Suzuki stage on admission, and rate of incision healing.

### Surgical Modalities

STA-MCA bypass was performed on the symptomatic and hemodynamically affected hemisphere. This kind of procedure involves end-to-side anastomosis of the branches of the STA to the cortical branches of the MCA. Briefly, the anterior branch of the STA was carefully dissected from the scalp flap, and the surrounding connective tissues were freed. Then, the STA segment was flushed with heparinized saline and set aside. A small craniotomy (diameter 3–5 cm) was performed over the Sylvian fissure. The dura was opened in a cruciate fashion, M4 branch of the MCA was selected as the recipient artery, and end-to-side anastomosis was performed. The immediate bypass patency was routinely confirmed with indocyanine green angiography.

### Perioperative Management and Evaluation

Patients underwent follow-up at the clinic or by telephone interview 3–6 months after surgery and annually thereafter. Follow-up DSA was conducted 3–12 months after the operation and reviewed by doctors blinded to patients’ groups. The primary follow-up event was bypass efficacy. Bypass efficacy was defined in this study as an integrated measure of overall bypass efficacy by DSA and measured with two indicators: bypass patency and postoperative neoangiogenesis. Postoperative neoangiogenesis was classified into three groups according to the criteria proposed by Matsushima et al. (1992). This grading was the proportion of the MCA area of distribution supplied by surgical revascularization: grade A, more than 2/3 of the MCA distribution; grade B, between 2/3 and 1/3 of the MCA distribution; and grade C, slight or none. An A or B score on Matsushima grading was defined as good or favorable neoangiogenesis, and a C score on Matsushima grading was defined as poor postoperative collateral formation. Then, the evaluation of Matsushima grading and patency was aggregated into a combined quality. Bypass efficacy was described as “good” if the Matsushima grading was scored as A/B, and patency was confirmed in the same case. Bypass efficacy was described as ‘poor’ if a ‘C’ or failure in patency occurred in the case. The secondary outcomes were the occurrence of 1) cerebral ischemic events such as ischemic stroke and TIA; 2) major/minor bleeding complications, defined as cerebral/gastrointestinal, skin, and mucosal hemorrhage; and 3) mortality. The mRS score at the last follow-up was used to evaluate the recovery status, which was classified as good (mRS score ≤2) or poor (mRS score ≥3).

### Statistical Analysis

The propensity score (PS) was calculated for each patient using a multivariable logistic regression model with covariates of sex, age, risk factors, individual comorbidities, symptoms, hospitalization days, mRS at admission, Suzuki stage, and rate of incision healing, with aspirin versus non-aspirin therapy as a binary-dependent variable. Pairs of patients were derived using greedy 1:1 matching (https://cran.r-project.org/web/packages/Matching). The quality of the match was assessed using the standardized mean difference, for which an absolute standardized difference >10% is suggested to represent a meaningful covariate imbalance. Associations between postoperative aspirin therapy and primary/secondary outcomes were estimated by logistic regression and presented as odds ratios (ORs) and 95% CIs. Multivariable logistic regression analysis was performed to identify the variables associated with the primary outcomes. Cumulative incidence was computed and compared between different groups obtained from the life table using the Kaplan–Meier approach (https://cran.r-project.org/web/packages/survival). A p-value <0.05 was considered to indicate statistical significance. Associations between bypass efficacy and clinical outcomes were evaluated using the phi coefficient with the following ordinal scale: strong negative (−1.0 to −0.7); weak negative (−0.7 to −0.3); little or no (−0.3 to 0.3); weak positive (0.3–0.7); and strong positive (0.7–1.0). All statistical analyses were performed using R statistical software (version 4.1.2; R Foundation for Statistical Computing, Vienna, Austria).

## Results

**FIGURE 2:**
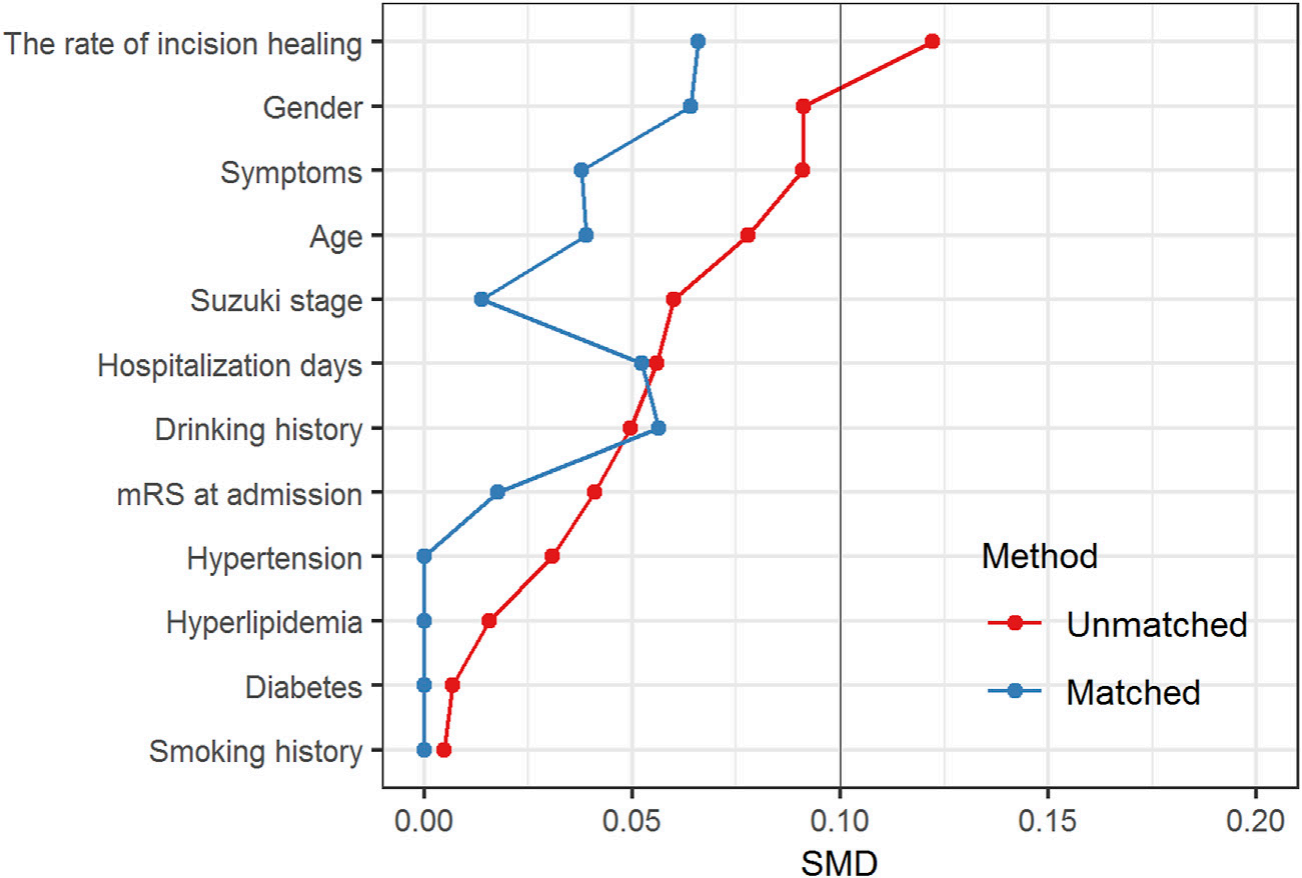
Standardized mean difference (SMD) for baseline covariates before and after PS matching. SMD >10% is considered to represent covariate imbalance.

A total of 201 STA-MCA procedures performed on 181 patients comprised the entire cohort. Among these procedures, 95 and 106 were discharged on aspirin (100 mg once a day) or without antiplatelet therapy, respectively. The baseline characteristics of the two groups are presented in Table 1. Propensity score (PS) matching was successful for 95 matched pairs of procedures performed with or without postoperative aspirin administration. After PS matching, the two groups were comparable for all pretreatment variables investigated (Figure 2).

**TABLE 1:** Baseline characteristics of patients in the aspirin and non-aspirin groups.

	Unmatched			Matched		
Characteristic	Non-aspirin	Aspirin	SMD	Non-aspirin	Aspirin	SMD
N	106	95	—	95	95	
Gender,n (%)	—	—	0.091	—	—	0.064
Male	41 (38.7)	41 (43.2)	—	38 (40.0)	41 (43.2)	—
Female	65 (61.3)	54 (56.8)	—	57 (60.0)	54 (56.8)	—
Age, years, mean (SD)	45.75 (9.81)	45.01 (9.30)	0.078	45.38 (9.57)	45.01 (9.30)	0.039
Risk factors, n (%)	—	—	—	—	—	—
Smoking history	27 (25.5)	24 (25.3)	0.005	24 (25.3)	24 (25.3)	<0.001
Drinking history	17 (16.0)	17 (17.9)	0.049	15 (15.8)	17 (17.9)	0.056
Individual comorbidities, n (%)	—	—	—	—	—	—
Hypertension	35 (33.0)	30 (31.6)	0.031	30 (31.6)	30 (31.6)	<0.001
Hyperlipidemia	2 (1.9)	2 (2.1)	0.016	2 (2.1)	2 (2.1)	<0.001
Diabetes	8 (7.5)	7 (7.4)	0.007	7 (7.4)	7 (7.4)	<0.001
Symptoms, n (%)	—	—	0.091	—	—	0.038
Stroke	58 (54.7)	53 (55.8)	—	54 (56.8)	53 (55.8)	—
TIA	35 (33.0)	33 (34.7)	—	33 (34.7)	33 (34.7)	—
Others	13 (12.3)	9 (9.5)	—	8 (8.4)	9 (9.5)	—
Hospitalization days, mean (SD)	18.76 (5.62)	19.12 (6.87)	0.056	18.79 (5.55)	19.12 (6.87)	0.052
mRS at admission (%)	—	—	0.041	—	—	0.018
0	12 (11.3)	9 (9.5)	—	8 (8.4)	9 (9.5)	—
1	71 (67.0)	65 (68.4)	—	66 (69.5)	65 (68.4)	—
2	21 (19.8)	19 (20.0)	—	19 (20.0)	19 (20.0)	—
3	2 (1.9)	2 (2.1)	—	2 (2.1)	2 (2.1)	—
Suzuki stage, n (%)	—	—	0.060	—	—	0.014
1	1 (0.9)	1 (1.1)	—	1 (1.1)	1 (1.1)	—
2	18 (17.0)	13 (13.7)	—	15 (15.8)	13 (13.7)	—
3	46 (43.4)	43 (45.3)	—	40 (42.1)	43 (45.3)	—
4	40 (37.7)	37 (38.9)	—	38 (40.0)	37 (38.9)	—
5	1 (0.9)	1 (1.1)	—	1 (1.1)	1 (1.1)	—
Rate of incision healing, n (%)	100 (94.3)	92 (96.8)	0.122	93 (97.9)	92 (96.8)	0.066

SMD, standardized mean difference; SD, standard deviation; TIA, transient ischemic attack; mRS, modified Rankin scale.

### Aspirin Use and Bypass Efficacy

After a mean follow-up of 2.7 years, 80 and 15 cases were classified as grades A/B and C, respectively, in the aspirin group; 68 and 27 cases were classified as grades A/B and C, respectively, in the non-aspirin group according to the Matsushima neoangiogenesis grading system (Table 2). Compared with non-use, postoperative use of aspirin was associated with favorable Matsushima grading scores (A/B) among patients with STA-MCA procedures [odds ratio (OR) 2.12, 95% confidence interval (CI) 1.05–4.39; *p* = 0.038].

**TABLE 2:** Bypass efficacy in matched patients with aspirin or non-aspirin therapy.

Outcome	Non-aspirin	Aspirin	OR (95%CI)	P
N	95	95	—	—
Matsushima stage, n (%)	—	—	2.12 [1.05, 4.39]	0.038
A + B	68 (71.6)	80 (84.2)	—	—
C	27 (28.4)	15 (15.8)	—	—
Bypass patency, n (%)	86 (90.5)	93 (97.9)	4.87 [1.21, 32.51]	0.047
Bypass efficacy, n (%)	—	—	2.23 [1.11, 4.61]	0.026
Good	67 (70.5)	80 (84.2)	—	—
Poor	28 (29.5)	15 (15.8)	—	—

OR, odds ratio; CI, confidence interval.

In addition, we observed a statistically significant difference in the patency rates between the two PS matching groups. Patients who were treated with aspirin had a higher rate of bypass patency than those without aspirin (97.9 vs. 90.5%, *p* = 0.047). Overall, patients in the aspirin group presented a significantly higher incidence of good bypass efficacy than those in the non-aspirin group [OR 2.23, 95% CI 1.11–4.61; *p* = 0.026].

The results of the logistic multivariate analysis of the factors affecting bypass efficacy are shown in Table 3. The adjusted multiple logistic regression model confirmed postoperative aspirin as an independent predictor of bypass patency, collateral formation, and overall bypass efficacy [adjusted OR 2.91, 95% CI 1.34–6.68; *p* = 0.009]. Suzuki stage, prior TIA, and the rate of incision healing also played roles in influencing bypass efficacy (*p* < 0.05).

**TABLE 3:** Association of patient features with bypass efficacy.

	Bypass efficacy			
Variable	Crude OR (95%CI)	P	Adjusted OR (95%CI)	P
Age	1.02 [0.98, 1.06]	0.259	1.02 [0.98, 1.06]	0.29
Diabetes	1.08 [0.32, 4.94]	0.911	0.76 [0.19, 3.96]	0.722
Drinking history	0.58 [0.26, 1.39]	0.205	0.57 [0.14, 2.18]	0.421
Aspirin	2.23 [1.11, 4.61]	0.026	2.91 [1.34, 6.68]	0.009
Gender/female	1.30 [0.65, 2.57]	0.456	0.98 [0.35, 2.56]	0.961
Hospitalization days	1.00 [0.95, 1.06]	0.977	1.01 [0.95, 1.08]	0.694
Hyperlipidemia	/	/	/	/
Hypertension	1.45 [0.69, 3.24]	0.338	1.48 [0.62, 3.79]	0.391
mRS at admission	1.15 [0.65, 2.08]	0.647	1.37 [0.70, 2.77]	0.37
Smoking history	0.54 [0.26, 1.15]	0.102	0.49 [0.15, 1.61]	0.227
Suzuki stage	0.74 [0.46, 1.18]	0.218	0.49 [0.26, 0.87]	0.019
Symptoms/stroke	0.71 [0.11, 2.82]	0.662	0.62 [0.08, 3.02]	0.595
Symptoms/TIA	0.23 [0.03, 0.92]	0.067	0.14 [0.02, 0.68]	0.03
Rate of incision healing	5.44 [0.87, 42.35]	0.069	9.59 [1.12, 102.15]	0.04

Adjusted OR, adjusted odds ratio, which was retrieved *via* multivariable logistic regression; CI, confidence interval; mRS, modified Rankin scale; TIA, transient ischemic attack.

### Aspirin Use and Postoperative Complications

**FIGURE 3:**
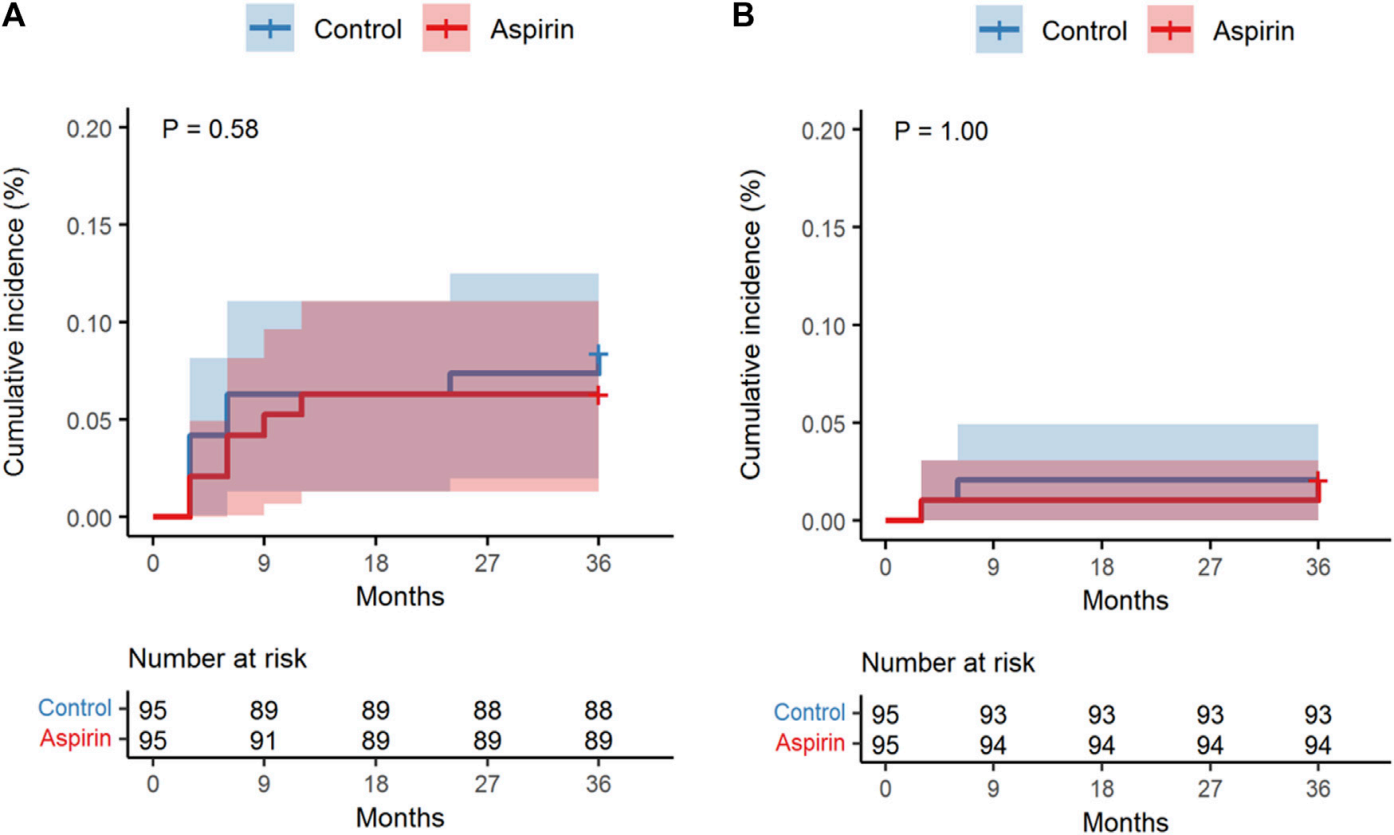
Kaplan–Meier curve showing the cumulative incidence of ischemic complications **(A)** and bleeding complications **(B)** between the aspirin and control groups in the entire cohort.

Postoperative complications in the 95 PS-matched pairs are presented in Table 4. The rate of cerebral ischemic events was six (6.3%) and eight (8.4%) in the aspirin and non-aspirin groups, respectively. There was a trend that postoperative aspirin therapy was related to improved clinical outcomes, but the difference was not statistically significant [OR 0.73, 95% CI 0.23–2.19; *p* = 0.580; Figure 3A]. We found no substantial differences when comparing the bleeding risk among users of postoperative aspirin with that among non-users (Figure 3B).

**TABLE 4:** Risk of ischemic complications and bleeding events with postoperative drug therapy.

Outcome	Non-aspirin	Aspirin	OR (95%CI)	P
N	95	95	—	—
Ischemic complications, n (%)	8 (8.4)	6 (6.3)	0.73 [0.23, 2.19]	0.580
Ischemic stroke, n (%)	5 (5.3)	4 (4.2)	0.79 [0.19, 3.08]	0.733
Post TIA, n (%)	3 (3.2)	2 (2.1)	0.66 [0.09, 4.07]	0.653
Mortality, n (%)	2 (2.1)	0 (0.0)	—	—
Bleeding complications, n (%)	2 (2.1)	2 (2.1)	1.00 [0.12, 8.48]	1.000
Major bleeding, n (%)	0 (0.0)	1 (1.1)	—	—
Minor bleeding, n (%)	2 (2.1)	1 (1.1)	0.49 [0.02, 5.25]	0.568
Other complications, n (%)	—	—	—	—
Asthma	2 (2.1)	1 (1.1)	0.49 [0.02, 5.25]	0.568
Seizure	5 (5.3)	9 (9.5)	1.88 [0.62, 6.34]	0.273
Follow-up mRS, n (%)	—	—	0.13 [0.01, 0.77]	—
0–2	88 (92.6)	94 (98.9)	—	0.062
3–6	7 (7.4)	1 (1.1)	—	—

OR, odds ratio; CI, confidence interval; TIA, transient ischemic attack; mRS, modified Rankin scale.

Major bleeding complications: cerebral hemorrhage.

Minor bleeding complications: gastrointestinal, skin, and mucosal hemorrhage.

### Associations Between Bypass Efficacy and Clinical Outcomes

**FIGURE 4:**
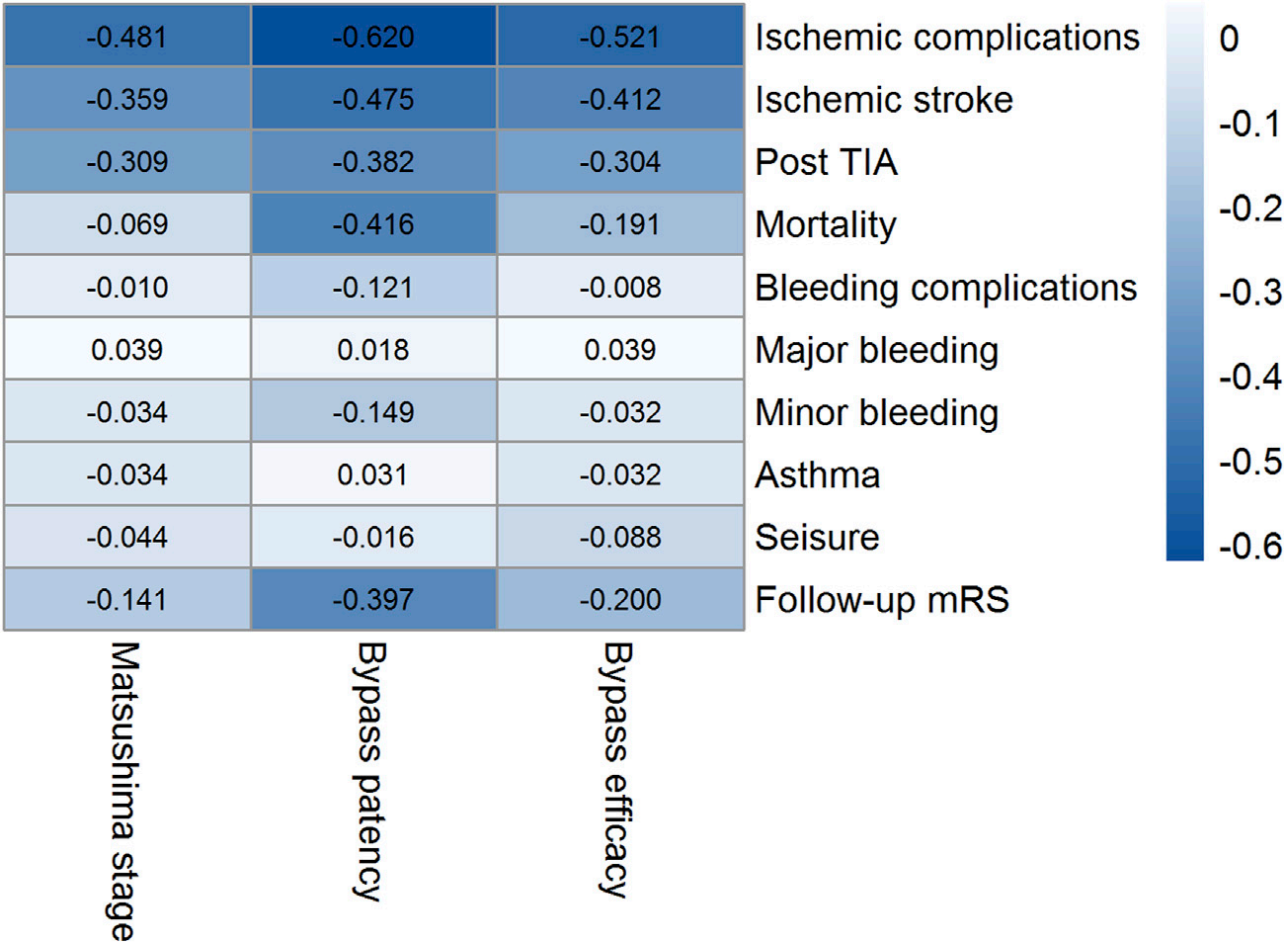
Correlogram of bypass efficacy and postoperative complications by study visit. Correlations were strong between the level of bypass efficacy and the rate of ischemic complications.

Associations between bypass efficacy and clinical outcomes were examined by using phi coefficients (Figure 4). A negative association was present between overall bypass efficacy and the occurrence of ischemic complications (Phi = −0.521). We also observed correlations between ischemic complications and the two indicators of bypass efficacy, Matsushima grading (Phi = −0.481) and patency (Phi = −0.620). Overall bypass efficacy was not statistically associated with the rate of bleeding complications (Phi = −0.008).

## Discussion

The relationship between STA-MCA bypass efficacy in patients with moyamoya disease, including bypass patency and postoperative neoangiogenesis, and clinical outcomes were compared between aspirin and non-aspirin groups.

Donaghy and Yasargil (1968) introduced the STA-MCA bypass in 1969 and initiated the practice of bypass surgery. STA-MCA bypass has gained in popularity because it provides an immediate increase in perfusion for ischemic MMD patients (Guzman et al., 2009; Gazyakan et al., 2015). Furthermore, the long-term prognosis of STA-MCA bypass is associated with postoperative revascularization, which is a complicated progression (Arias et al., 2014; Arias et al., 2015).

Although studies have indicated that both bypass patency and postoperative neoangiogenesis contribute to revascularization in adult patients with ischemic MMD, limited attention has been devoted to the combined impact of patency and neoangiogenesis on the clinical outcomes of MMD patients after STA-MCA bypass (Liu et al., 2019; Lu et al., 2021). The Matsushima neoangiogenesis grading system has been widely reported to be applied in patients undergoing indirect or combined bypass (Matsushima and Inaba, 1986; Chen et al., 2020) but not in those undergoing direct bypass. However, we have accumulated a large amount of experience with Matsushima grading for evaluating the efficacy of STA-MCA bypass. In this study, we integrated two indicators, patency and neoangiogenesis, to propose a new measure for bypass efficacy assessment with a definition of good or poor that correlates well with the therapeutic outcome.

In the present study, a strong negative association was found between the overall bypass efficacy and the rate of ischemic complications. The two indicators of bypass efficacy, Matsushima grading and patency, were also correlated with the incidence of ischemic complications (Figure 4). These results support the use of our combined measure of bypass efficacy because we found a favorable bypass efficacy was associated with better outcomes of the STA-MCA procedures.

Although safe and elegant revascularization techniques have been developed and refined to augment perfusion, whether an antiplatelet regimen is appropriate after revascularization remains an area of controversy. Ideal antiplatelet therapy should be effective in improving symptoms, preventing recurrent stroke, and keeping the bleeding risk at low levels. Currently, related studies have provided conflicting results on the effects of aspirin on the clinical prognosis post revascularization. Some retrospective studies have observed that postoperative aspirin use might be beneficial for maintaining blood flow of the bypass and preventing thromboembolic events (Kraemer et al., 2012; Lu et al., 2020; Lu et al., 2021). However, other studies have pointed out that aspirin does not improve ischemic symptoms and may increase the risk of intracranial hemorrhage (Kraemer et al., 2012; Yamada et al., 2016).

Based on the present study, postoperative aspirin administration does not appear to significantly increase the rate of bleeding risk following surgery (Figure 3B). On the other hand, among subjects administered aspirin, we found a non-significant trend toward a larger protective effect from ischemic complications (Figure 3A). Aspirin therapy was associated with a decreased risk of mortality, but it did not reach statistical significance, mainly due to the small number of patients in each group (Table 4). Our study also found a potential role for aspirin in patency improvement and neoangiogenesis development over a prolonged period. Compared with non-users, postoperative users of aspirin had improved overall bypass efficacy during follow-up in this cohort (Table 2). Furthermore, the logistic regression multivariate analysis confirmed the benefit of aspirin when bypass efficacy was taken into account (Table 3). Based on the phi coefficient analysis, we have demonstrated that overall bypass efficacy is essential to determine the success of the STA-MCA procedures as well as the long-term prognosis of patients receiving cerebrovascular bypass.

We concluded that postoperative aspirin therapy is associated with higher patency and more favorable Matsushima neoangiogenesis grading scores, which can in turn affect long-term outcomes in adult patients undergoing STA-MCA procedures.

## Limitation

The major limitation of the present study is its single-center, retrospective nature. We used propensity score matching to overcome any potential bias arising from unbalanced covariates; however, subject to the inherent bias of patient selection, the cases in our study may not be representative of the overall patient population. Our study analyzed bypass efficacy by aggregating Matsushima grading and patency into a combined outcome, but other factors contributing to bypass efficacy, including postoperative changes in moyamoya vessels, Suzuki stage, prior TIA, rate of incision healing, technical factors, and medications besides aspirin, should be kept in mind. The total number of postoperative complications was rather small during follow-up, and hence, sparse data bias could not be ruled out. Taken together, future investigations with randomized controlled designs and multicenter populations with larger sample sizes are needed.

## Conclusion

In this cohort, bypass efficacy was strongly correlated with ischemic event rates; the postoperative use of aspirin was associated with favorable bypass efficacy and improved postoperative outcomes of MMD after STA-MCA bypass procedures. Large prospective randomized controlled trials addressing the role of aspirin after STA-MCA bypass are urgently needed to provide more definitive guidance for clinicians.

## Data Availability

The original contributions presented in the study are included in the article/Supplementary Material, further inquiries can be directed to the corresponding authors.
